# *Origanum vulgare* L. Essential Oil as a Potential Anti-Acne Topical Nanoemulsion—In Vitro and In Vivo Study

**DOI:** 10.3390/molecules23092164

**Published:** 2018-08-28

**Authors:** Mohammed H. Taleb, Nourtan F. Abdeltawab, Rehab N. Shamma, Sherein S. Abdelgayed, Sarah S. Mohamed, Mohamed A. Farag, Mohammed A. Ramadan

**Affiliations:** 1 Department of Microbiology and Immunology, Faculty of Pharmacy, Cairo University, Cairo 11562, Egypt; almataleb21@gmail.com (M.H.T.); m_ramdan56@hotmail.com (M.A.R.); 2 Department of Pharmaceutics and Pharmaceutical Technology, Faculty of Pharmacy, Al-Azhar University–Gaza, PO Box 1277, Gaza 79702, Palestine; 3 Department of Pharmaceutics and Industrial pharmacy, Faculty of Pharmacy, Cairo University, Cairo 11562, Egypt; rehab.shamma@pharma.cu.edu.eg; 4 Department of Pathology, Faculty of Veterinary Medicine, Cairo University, Cairo 12211, Egypt; sherein.abdelgayed@vet.cu.edu.eg; 5 Department of Pharmacology and Toxicology, Faculty of Pharmacy, Cairo University, Cairo 11562, Egypt; sarah.elsayed@pharma.cu.edu.eg; 6 Department of Pharmacognosy, Faculty of Pharmacy, Cairo University, Cairo 11562, Egypt; mfarag73@yahoo.com; 7 Department of Chemistry, School of Sciences & Engineering, The American University in Cairo, Cairo 11853, Egypt

**Keywords:** acne vulgaris, essential oils, oregano, thymol, *S. epidermidis*, *P. acnes*, antimicrobial activity, nanoemulsion, antibiotic resistance

## Abstract

Antibiotics are often prescribed in acne treatment; however, *Propionibacterium acnes* and *Staphylococcus epidermidis*, the two of the major acne-associated bacteria, developed antibiotic resistance. Essential oils (EOs) present a natural, safe, efficacious and multifunctional alternative treatment. This study aimed to assess the potential anti-acne activity of selected seven EOs commonly used in Mediterranean folk medicine. Antimicrobial activity screening of these oils showed oregano to exhibit the strongest antimicrobial activity with minimum inhibitory concentration (MIC) of 0.34 mg/mL and minimum bactericidal concentration (MBC) of 0.67 mg/mL against *P. acnes*; and MIC of 0.67 mg/mL and MBC of 1.34 mg/mL against *S. epidermidis*. The composition of the most effective EOs (oregano and thyme) was determined using gas chromatography-mass spectrometry (GC-MS). Monoterpenoid phenols predominated oregano and thyme EO with thymol percentile 99 and 72, respectively. Thymol showed MIC 0.70 mg/mL against both *P. acnes* and *S. epidermidis* whereas MBC was 1.40 and 2.80 mg/mL against *P. acnes* and *S. epidermidis*, respectively. Moreover, oregano exhibited the strongest anti-biofilm effect against *S. epidermidis* with MBIC 1.34 mg/mL and killing dynamic time of 12 and 8 h against *P. acnes* and *S. epidermidis*, respectively. Oregano, the most effective EO, was formulated and tested as a nanoemulsion in an acne animal mouse model. The formulation showed superior healing and antimicrobial effects compared to the reference antibiotic. Collectively, our data suggested that oregano oil nanoemulsion is a potential natural and effective alternative for treating acne and overcoming the emerging antibiotic resistance.

## 1. Introduction

Acne vulgaris is the 8th most prevalent disease and second top skin disease globally [1]. Adolescents of both genders are typically affected by acne. Acne in adolescents can cause psychological disorders and in severe cases can lead to depression [2]. Acne is a multifactorial disease characterized by pathological alteration in pilosebaceous units of the neck and upper trunk. It results in the formation of non-inflammatory comedones and inflammatory lesions such as papules, pustules, and nodules [3]. Two bacteria are associated with acne pathogenesis: *Propionibacterium acnes and Staphylococcus epidermidis* [4]. These bacteria are part of normal human skin microbiota, but if dysbiosis occurs, infection of pilosebaceous units can lead to acne. Accordingly, most anti-acne drugs are directed against *P. acnes* and *S. epidermidis* infection and associated inflammatory responses. Anti-acne therapy includes systemic and topical therapies. Topical therapies include comedolytic agents, anti-inflammatory agents and antibiotics [5]. Bacterial resistance accompanies topical anti-acne antibiotics. Resistance to topical anti-acne antibiotics is attributed to multiple factors, including use of repeated single antibiotic, sub-inhibitory concentrations, or use over extended time [6,7].

To overcome the emerging resistance to conventional antibiotics, alternative natural antimicrobial agents have been investigated. Essential oils (EOs) of aromatic plants such as oregano, tea tree oil, lemongrass, and thyme have antimicrobial activities that can be used as a natural alternative [8]. The antimicrobial activity of these EOs is attributed to their major constituents: Monoterpenoid phenols. In addition, EOs’ minor constituents, such as the monoterpene hydrocarbons, γ-terpinene, and *p*-cymene, may contribute to the antibacterial activity of these oils [9,10]. Some studies show that a few EOs have anti-acne activity; tea tree oil is commercially used [11]. Therefore, this study tested the anti-acne potential of seven EOs used in Mediterranean folk medicine: Oregano (*Origanum vulgare*), thyme (*Thymus vulgaris*), lemongrass (*Cymbopogon citratus*), tea tree (*Melaleuca alternifolia*), mentha (*Mentha piperita*), lavender (*Lavendula anguestifolia*), and chamomile (*Matricaria recutita*). The Mediterranean region is one of the largest producers of these aromatic plants. Its favorable climatic and cultivation conditions enhance the quality of EO constitutes [12]. We assessed the antimicrobial activity of the above-selected EOs against acne-causing bacteria in vitro.

Another aim of this study was to develop a pharmaceutical formulation of the EO with the highest antimicrobial effect. Based on our in vitro antibacterial results of tested EOs against acne-causing bacteria, we formulated oregano EO as a nanoemulsion formula. Nanoemulsions offer enhanced solubilization capacity for hydrophobic, poorly soluble drugs as typical in the case of EOs [13]. In this study, we assessed the healing and antimicrobial activity of the developed nanoemulsion of the most effective EO in vivo in an acne mouse model as a potential new formulation for acne treatment.

## 2. Results

### 2.1. Screening of Antibacterial Activity of Tested EOs Using Agar Disc Diffusion Method

**Figure 1:**
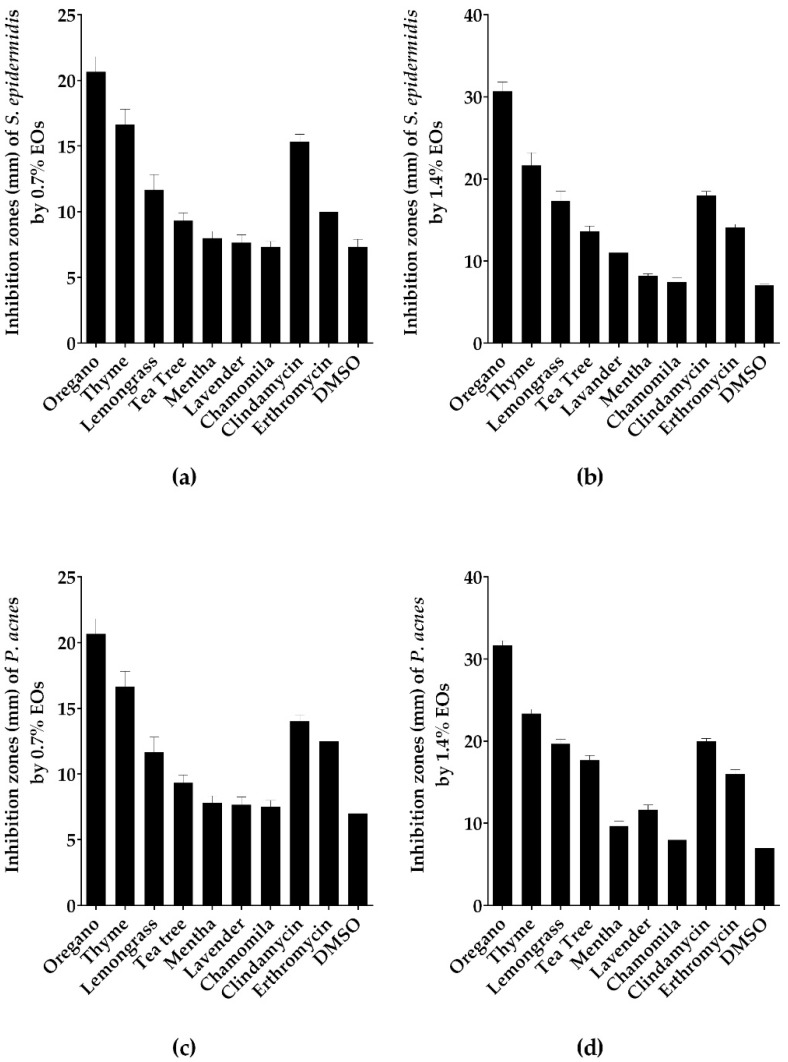
Oregano EO exhibited the largest inhibition zone among the tested oils. Antibacterial activity of the screened EOs using agar disc diffusion method against (**a**) *S. epidermidis* with EOs at concentration of 0.7%; (**b**) *S. epidermidis* with EOs at concentration of 1.4%; (**c**) *P. acnes* with EOs at concentration of 0.7%; (**d**) *P. acnes* with EOs at concentration of 1.4%. Data is represented as means of inhibition zones (mm) ± SD. Controls used were clindamycin and erythromycin as positive control, while DMSO as negative control.

The selected seven EOs have been screened for their antibacterial activities against *P. acnes* and *S. epidermidis* using the disc diffusion test. The results were represented as the diameter of inhibition zone (Figure 1 and Table S1). *P. acnes* and *S. epidermidis* were susceptible to oregano and thyme EOs with a zone of inhibition (ZOI) ranging from 16 to 32 mm larger than those of erythromycin and clindamycin reference antibiotics (Figure 1). While lemongrass and tea tree showed moderate ZOI from 9–17 mm, lavender, mentha, chamomile EOs failed to inhibit the growth of both bacteria (Figure 1 and Table S1). The two studies’ positive controls (erythromycin and clindamycin) showed ZOI ranging from 10 to 20 mm. The negative control vehicle dimethylsulfoxide (DMSO) showed no inhibition against both bacteria.

### 2.2. Antimicrobial Activity of the Tested EOs Using Minimal Inhibitory Concentration (MIC) Method

Minimal inhibitory concentration (MIC) test results confirmed that the most potent EOs against *P. acnes* and *S. epidermidis* were oregano, thyme, lemongrass and tea tree oils (Table 1). Oregano EO displayed lowest MIC values of 0.34, 0.67 mg/mL against *P. acnes* and *S. epidermidis*, respectively. Second to oregano was thyme EO, exhibiting an MIC of 0.65, 1.30 mg/mL against *P. acnes* and *S. epidermidis*, respectively. Consequentially, oregano, thyme, lemongrass, and tea tree EOs were selected for determining minimum bactericidal concentration (MBC), demonstrating the most effective inhibition against *P. acnes* and *S. epidermidis* by disc diffusion and MIC assays.

### 2.3. The Bactericidal and Anti-Biofilm Activities of the Tested EOs

The bactericidal activity of the four EOs with the top ZOIs and MICs were tested against acne-causing bacteria. Oregano EO at a concentration of 0.67 and 1.34 mg/mL inhibited the growth of *P. acnes and S. epidermidis*, respectively (Table 2). Tea tree EO exhibited the least bactericidal activity at 2.558 and 5.116 mg/mL against *P. acnes* and *S. epidermidis*, respectively. Next, the four-selected EOs were assayed for anti-biofilm activity against *S. epidermidis* (Table 3). Oregano EO inhibited biofilm formation by *S. epidermidis* at its bactericidal concentration (1.34 mg/mL). In contrast, the least effective EO was tea tree oil was unable to inhibit biofilm formation of *S. epidermidis* at concentration up to 2.56 mg/mL equivalent to 0.28% *v*/*v* (Table 3).

### 2.4. Killing Dynamics of Oregano EO against S. epidermidis and P. acnes

**Figure 2:**
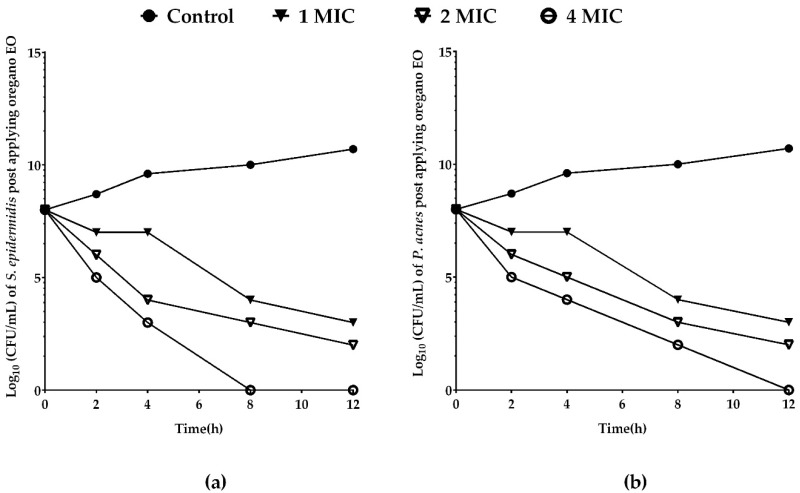
Oregano EO inhibited the growth of *S. epidermidis* faster than *P. acnes*. Oregano EO at concentrations of 0.672, 1.34, 2.68 mg/mL (1, 2, 4 MIC) and no EO control was used for assaying the killing rate of bacterial cells at 0, 2, 4, 8 and 12 h and expressed as the surviving bacteria (log_10_ CFU/mL) at different exposure times. We used starting bacterial suspension concentration 10^8^ CFU/mL. Oregano EO killing rate against (**a**) *S. epidermidis* and (**b**) *P. acnes.*

Since our results of agar disc diffusion, MIC, MBC and MBIC assays showed that oregano EO was superior, the dynamics of oregano EO’s killing activity was determined. MIC, 2MIC and 4MIC of oregano EO showed significant reduction in the number of both *P. acnes* and *S. epidermidis* after 4 h of application (Figure 2). Oregano EO killing capacity against *S. epidermidis* was higher than *P. acnes* at 8 and 12 h (Figure 2). Oregano EO volatile constituents were determined using gas chromatography-mass spectrometry (GC-MS) analysis to pinpoint active agents in oregano EO likely to mediate the observed anti-acne effects.

### 2.5. Chemical Composition of Oregano and Thyme EOs

Both oregano and thyme belong to the same family (*Lamiaceae*), producing aromatics as major essential oil constituents. Our GC-MS analysis of oregano and thyme EO volatile constitutes showed that the major volatile compound was thymol (Table 4 and Figure S1). Thymol represented 99.44% of oregano EO and 72% of thyme volatile compounds. Other volatiles identified were at much-lower levels in both oils and include *p*-cymene, cineole and γ-terpinene (Table 4).

The antimicrobial activity data showed that oregano EO exhibited the largest inhibition zones and MIC among the tested seven oils. Oregano EO displayed the best MBC and MBIC among selected top four EOs, along with rapid killing rates of both tested bacteria. Moreover, our GC-MC analysis showed that thymol content in oregano EO was higher than thyme EO, suggesting that thymol might be the reason for oregano superior antimicrobial activities. Therefore, we next aimed to assay thymol antimicrobial activity compared to oregano and thyme EOs.

### 2.6. Antimicrobial Activity of Thymol Using In Vitro Disc Diffusion, MIC, MBC and MBIC Assays

**Figure 3:**
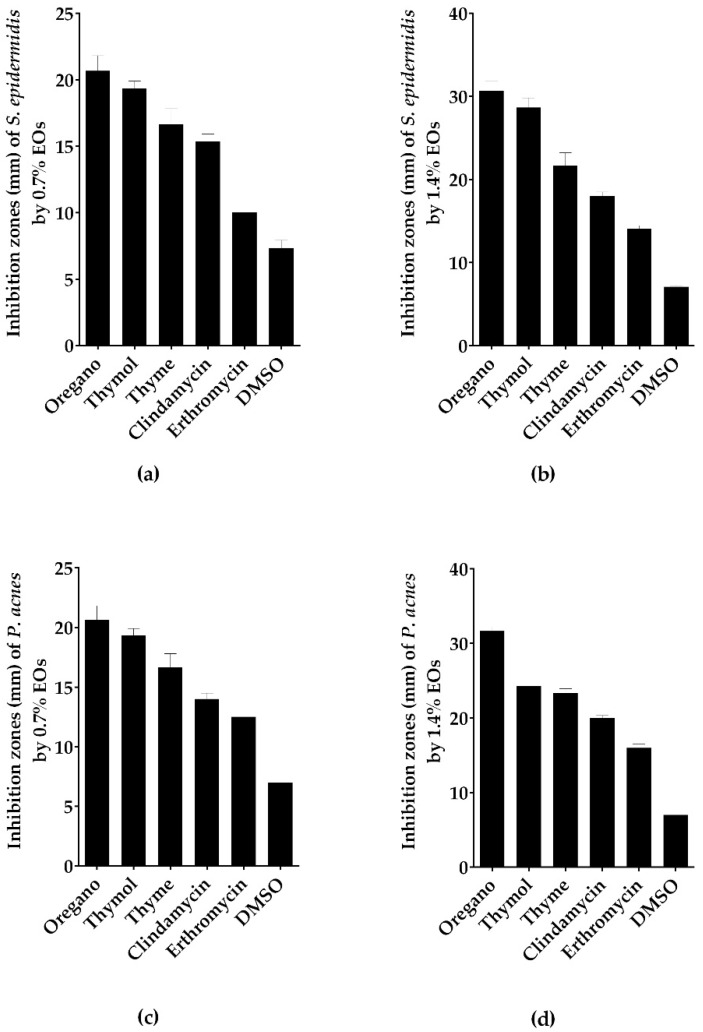
Thymol exhibited lower inhibition zone than oregano EO. Antibacterial activity of thymol and the most bioactive EOs using disc diffusion method (zone of inhibition in mm) against (**a**) *S. epidermidis* with thymol and EOs at concentration of 0.7%; (**b**) *S. epidermidis* with thymol and EOs at concentration of 1.4%; (**c**) *P. acnes* with thymol and EOs at concentration of 0.7%; (**d**) *P. acnes* with thymol and EOs at concentration of 1.4%. Data is represented as means of inhibition zones (mm) ± SD. Controls used were clindamycin and erythromycin as positive control with DMSO as negative control.

The antimicrobial activity of thymol, the main volatile constituent of oregano and thyme EOs, was assayed. Using the disc diffusion method, thymol at 0.7% and 1.4% concentration inhibited the growth of *S. epidermidis* and *P. acnes* significantly higher than erythromycin (inhibition zone > 19 mm) (Figure 1 and Figure 3, Table S1). On assaying of the MIC of thymol against *S. epidermidis* and *P. acnes,* oregano and thyme were found to be superior to thymol (Table 1 and Table 5)*.* Similarly, MBC of thymol against *P. acnes* was lower than oregano and thyme. MBC of thymol against *S. epidermidis* was intermediate between oregano and thyme EOs (Table 2 and Table 5). Finally, thymol antibiofilm activity against *S. epidermidis* was lower than oregano, thyme, lemongrass and tea tree EOs (Table 3 and Table 5).

Despite the predominance of thymol in oregano EO, in vitro antimicrobial assays showed that oregano EO was superior to thymol alone. Therefore, we selected oregano EO for nanoemulsion formulation and tested it in vivo as a topical anti-acne treatment.

### 2.7. Nanoemulsion Formulation of Oregano EO

EOs exhibit poor water solubility, hindering their application in aqueous formulations. O/W nanoemulsion of oregano EO was formulated to improve its poor water solubility. It contained less than 5% (*w*/*w*) of oregano EO and Pluronic F127, exhibiting a clear stable solution. The nano formulation stability was assessed for four weeks at room temperature. No signs of cloudiness, creaming or phase separation were observed. The prepared nanoemulsion had a particle size of 39.54 nm, with a polydispersity index of 0.285, indicating its low size distribution. Figure S2 shows the results of mean droplet size and polydispersity index of the prepared nanoemulsion.

### 2.8. Assessment of In Vivo Antimicrobial and Healing Activities of Oregano EO Nanoemulsion Using In Vivo Acne Mouse Model

**Figure 4:**
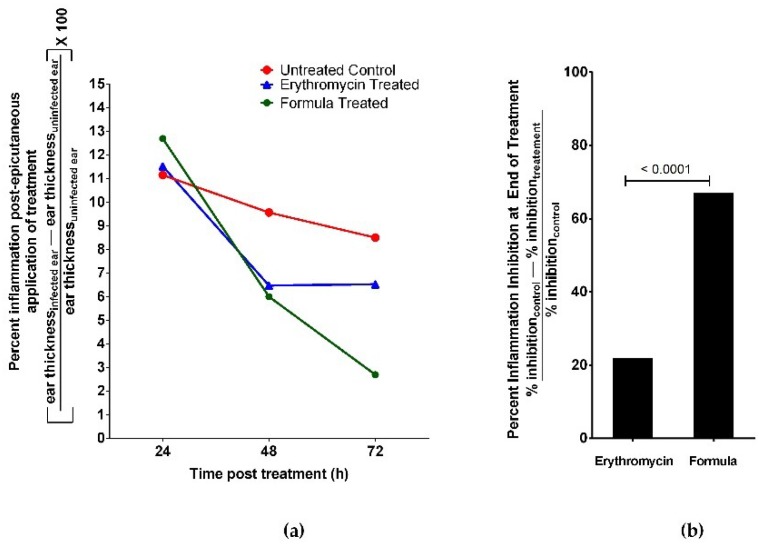
Oregano EO nanoemulsion showed stronger inhibition of inflammation than erythromycin control in acne mouse model. The acne mouse model was induced by intradermal injection of BALB/c mice’s left ears with 10^8^ CFU in 20 mL of *P. acnes*. The mice’s right ears served as uninfected control. We applied epicutanously either oregano EO nanoemulsion or 2% Erythromycin solution on the infected mice ears. A third group of mice served as untreated control. (**a**) Percent inflammation was assessed post epicutaneous application of treatment as the difference between each mouse ear thickness of infected and uninfected ears. Oregano nanoemulsion showed higher rate of reduction in inflammation than 2% erythromycin during the treatment time interval. (**b**) Oregano nanoemulsion showed significantly higher percent of inhibition of inflammation (>60%) at the end of treatment period than 2% erythromycin control (20%). Data represented as mean ± standard deviation of three independent experiments, total mice per group 15 mice. We used *t*-test for comparing groups with *p* < 0.05 consider significant.

Oil dose was adjusted in the formulation at 2 MIC as 0.672 mg/mL in order to avoid possible irritation or hypersensitive skin reaction. We assessed the anti-acne activity of the formulated nanoemulsion of oregano EO using in vivo acne mouse model. We infected BALB/c mice ears intradermally with *P. acnes*. After two days, nano formulation or 2% erythromycin epicutanously was applied on mice ears and measured anti-inflammatory and antimicrobial activity against *P. acnes*. A third group of mice was injected with phosphate buffered saline (PBS) without *P. acnes* as a healthy control group. Mice of the uninfected ear control group had no detectable bacterial growth. We also evaluated healing by examining histopathology of mouse ear tissue. We compared results to mice treated with 2% erythromycin (positive controls) and untreated mice (negative controls). Nanoemulsion of oregano EO significantly reduced inflammation at the end of the experiment (Figure 4). The percentage inhibition of inflammation for all animal groups was calculated using the following formula [14]:

$$\%\;\mathrm{inflammation}\;\mathrm{inhibition}=\frac{\%\mathrm{inhibition}\;\left({}_{\mathrm{control}}\right)-\%\mathrm{inhibition}\;\left({}_{\mathrm{treatment}}\right)}{\%\mathrm{inhibition}\;\left({}_{\mathrm{control}}\right)}$$

**Figure 5:**
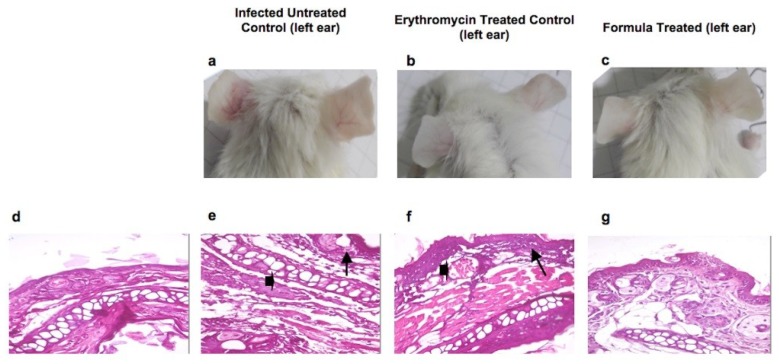
Oregano nanoemulsion as a potent anti-acne agent tested in vivo model of *P. acnes* infection Photo images of BALB/c mice ear skin at the end of the experiment showing (**a**) untreated control mice, one ear injected with 20 μL of 10^8^ CFU *P. acnes* suspension showing microcomedones and inflammation in infected left ear compared to the uninfected right ear; (**b**) inflammation in left infected ear disappeared after treatment with erythromycin solution comparable to the right uninfected ear; (**c**) absence of inflammatory appearance in left infected ear treated by oregano formula; (**d**) histopathological analysis of uninfected mice ear skin tissue stained with hematoxylin and eosin showing normal histology of mouse ear tissue with basal layer and epidermal cell maturation preserved; (**e**) histopathology of infected untreated control mice ear showing necrotic dermatitis character; note the severe dermal necrosis (arrow head), and lymphocytic infiltrate (arrow); (**f**) ear showing acute dermatitis character; note the congested blood vessels (arrow head), and slight lymphocytic cells infiltration (arrow) treated by erythromycin solution; (**g**) ear showing apparently normal histology of mouse ear tissue with the absence of inflammatory reaction, treated with oregano formula.

Inhibition of inflammation at the end of the experiment was significantly lower in mice treated with nanoemulsion compared to the positive control erythromycin (Figure 4). Moreover, the rate of reduction of mice ear thickness, post treatment, was also superior to erythromycin positive control (Figure 5).

**Figure 6:**
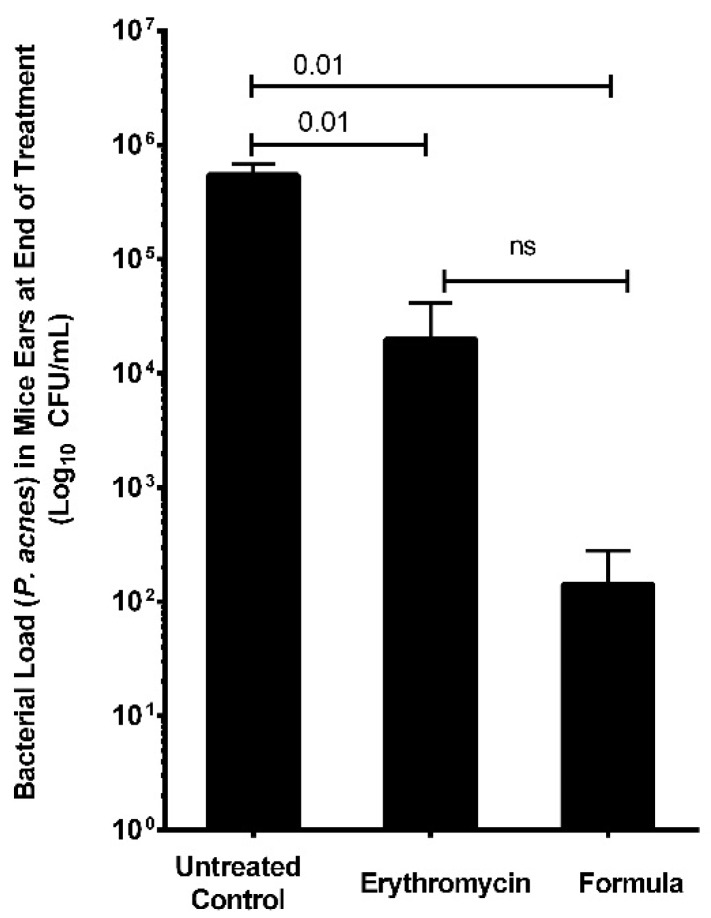
Oregano EO nanoemulsion showed higher antimicrobial effect against *P. acnes* than erythromycin control in acne mouse model. We induced acne in a mouse model by intradermal injection of BALB/c mice left ears with 10^8^ CFU in 20 µL of *P. acnes*. The mice’s right ears served as uninfected control. We applied epicutanously either oregano EO nanoemulsion or 2% Erythromycin solution on infected mice ears. A third group of mice served as untreated control. Viable counts of *P. acnes* load in mice ears at the end of the treatment of various mice groups were performed. Oregano nanoemulsion significantly lowered bacterial load in treated mice ears than 2% Erythromycin. Bacterial load was expressed as Log_10_ CFU/mL. Data represented as mean ± standard deviation of three independent experiments, total mice per group 15 mice. *T*-test was used for comparing groups with *p* < 0.05 considered significant.

The in vivo antimicrobial activity of oregano EO nanoemulsion was assessed where bacterial counts of *P. acnes* dropped from 1 × 10^8^ to 4.3 × 10^1^ CFU/mL post-treatment with 0.672 mg/mL of oregano nanoemulsion (Figure 6). In contrast, the bacterial number for the negative control group (treated with PBS buffer) was maintained at 5 × 10^5^ CFU/mL, whereas the positive control treated with erythromycin solution reduced the bacterial number to 3.5 × 10^3^ CFU/mL.

Finally, we assessed the healing effects of oregano EO nanoemulsion using histopathological and digital photography. Infected mice ears treated with nanoemulsion showed healing comparable to the erythromycin positive control, resembling normal ear tissue. Our in vivo results collectively suggested that oregano nanoemulsion effectively killed *P. acnes* and healed ear tissue inflammation better than erythromycin, the standard antibiotic for acne.

## 3. Discussion

Oregano EO exhibited the strongest antimicrobial effect against the tested acne-causing bacteria. Using disc diffusion, MIC, and MBC assays, oregano, thyme, and thymol were the top three antimicrobial agents against *P. acnes*. Meanwhile, tea tree and lemongrass EOs exhibited an intermediate antimicrobial effect against *P. acnes* and *S. epidermidis*. Both tested acne-causing bacteria were resistant to chamomile, lavender, and menthe EOs. Thus, in this study, the anti-acne effects of oregano EO surpassed that of other EOs evaluated including commercialized over-the-counter acne treatment tea tree EO [15,16]. Moreover, part of acne pathogenesis includes *S. epidermidis* biofilm formation. Bacteria growing in biofilms are more resistant to antibiotics compared to planktonic lifestyle [17]. Therefore, we assessed the anti-biofilm ability of the most potent antimicrobial EOs and found that oregano EO exhibited strongest anti-biofilm activity. Moreover, oregano EO at 4 MIC showed rapid killing of both tested acne-causing bacteria.

Thymol exhibited potent antimicrobial activity against acne-causing bacteria, but less than oregano EO itself. Thymol exhibits significant bactericidal activity [18,19,20] and reduces bacterial resistance to antibiotics [21]. Thymol antimicrobial action is mainly mediated via inhibiting bacterial growth and lactate production, decreasing cellular glucose uptake, causing lysis of fungal hyphal wall [22,23]. Thymol was the principal phenolic component of oregano EO (>99%) and thyme EO (>70%). It is thus suggested for its role in mediating the EOs’ antimicrobial effect. However, thymol alone was less potent than oregano EO. It could be that other minor volatiles present at lower levels such as *p*-cymene, γ-terpinene, α-thujene and cineole synergized thymol’s effect in oregano EO and may account for difference in activity between oregano and thymol. Composition of EOs depends on a number of factors, including harvesting seasons, plant cross-section, extraction method, and geographical sources (reviewed in [24]). This can account for differences between results obtained from different studies, where variation in thymol amount is significant [25,26]. The EOs used in this study were obtained from plants collected during the flowering stage, which could explain the high phenolic compound levels. Collectively, oregano EO was the most potent antimicrobial. Consequently, it was formulated in nanoemulsion topical dosage form to be assessed as anti-acne formula in vivo on acne-induced mouse model.

Treatment of the acne mouse model with proposed oregano nanoemulsion resulted in reduction of inflammation, bacterial load and healing of tissue superior to erythromycin. EOs’ antibacterial effects are improved through formulation as nanoemulsions [27,28,29]. The prepared emulsion had a particle size of 39.54 nm and a polydispersity index of 0.285, indicating its low size distribution; it is, therefore, considered as a nanoemulsion [30]. Pluronic F127 surfactant was used at a concentration of 4.5% and the oil at 0.5% *w*/*w*. The used concentration is above Pluronic F127 critical micelle concentration (CMC), where CMC of Pluronic F127 ranges between 0.26–0.8 wt%. Oregano oil exhibited anti-inflammatory, anti-leishmanial, antioxidant, hepatoprotective and anti-tumor activities, reviewed in [31]. To the best of our knowledge, this is the first study to report the anti-acne effect of oregano EO. Oregano’s anti-acne effects surpass those of the commercialized over-the-counter acne tea tree EO and select EOs with documented anti-microbial effects [11]. Most animals do not produce sufficient triglycerides to harbor *P. acnes* [32]. Therefore, we standardized our acne mouse model using BALB/c mice through intradermal injection of *P. acnes* in mice ears [32,33]. Based on our in vivo results, we propose BALB/c mice as another possible mouse strain for the development of an animal model of acne.

The essential oils of aromatic plants offer spasmolytic, mucolytic and cough soothing effects [31,34]. In addition, antimicrobial effects of EOs against pathogens and food contaminants have also been favored due to the safety of natural-origin products, preferred by the public [35]. Little attention, however, has been paid to EO’s antimicrobial effect against acne-causing bacteria. Overall, our results indicate that the proposed oregano nanoemulsion exhibits high antimicrobial and healing effects with fewer side-effects than anti-acne reference antibiotics. The potential use of our proposed oregano nanoemulsion as an anti-acne agent opens the field for new alternative treatments of natural origin, avoiding the problems associated with the use of antibiotics for acne. Further clinical studies on our proposed formulation can increase its potential as a drug to be used clinically in humans.

## 4. Materials and Methods

### 4.1. Essential Oils

Seven EOs were used in this study, viz., oregano (*Origanum vulgare*), thyme (*Thymus vulgaris*), lemongrass (*Cymbopogon citratus*), tea tree (*Melaleuca alternifolia*), mentha (*Mentha piperita*), lavender (*Lavendula anguestifolia*) and chamomile (*Matricaria recutita*). The pharmaceutical grade EOs were kindly provided as a gift from the Department of Food Science and Technology, Nebraska University, Lincoln, NE, USA. We determined the composition of oregano and thyme EOs by GC-MS analysis at the Department of Pharmacognosy, Faculty of Pharmacy, Cairo University, as detailed in Section 4.8. Pure thymol was purchased from Sigma Aldrich, St. Louis, MO, USA.

### 4.2. Bacterial Strains and Culturing

*Propionibacterium acnes* ATCC 6919 and *Staphylococcus epidermidis* ATCC 28319 were kindly provided by Dr. Mayri A. Diaz, Manchester University, UK. *S. epidermidis* was cultured aerobically on brain heart infusion (BHI) agar (LAB M limited, Lancashire, UK) and incubated at 37 °C for 18 h. We sub-cultured isolated colonies of *S. epidermidis* in BHI broth and incubated at 37 °C for 18 h aerobically. For *P. acnes*, we cultured the bacteria anaerobically on reinforced clostridial medium (RCM) agar (Oxoid Limited, Basingstoke, UK) for 48 h at 37 °C and ~5% CO_2_ using anaerobic jar and anaerobic atmosphere generation bags (Sigma-Aldrich, St. Louis, MO, USA). We sub-cultured isolated colonies of *P. acnes* in RCM broth for 48 h at 37 °C under anaerobic conditions.

### 4.3. Animals

Male BALB/c mice (6 weeks old, 20 g of weight) (*n* = 50 mice) were purchased from Theodor Bilharz Research Institute (Giza, Egypt). Research procedures were conducted in compliance with the principles and recommendations of the Guide for the Care and Use of Laboratory Animals Association, A.V.M. (2007) [36]. All animal experiments were approved by the research ethics committee of the Faculty of Pharmacy, Cairo University, protocol identification code (MI2002); date of approval: 30 May 2017.

### 4.4. Screening of Antibacterial Activities of EOs by Disc Diffusion Method

As a preliminary screening step, the antibacterial activities of all seven EOs was determined using agar disc diffusion, according to the Kirby-Bauer method [37] with some modification. EOs were diluted in analytical grade sterilized dimethylsulfoxide (DMSO) (Sigma Aldrich, St. Louis, MO, USA) and stock solutions of each of the oils at a concentration of 0.7% and 1.4% were prepared. We filter sterilized the prepared stock solutions by sterile syringe filter 0.2 µm (Corning, New York, NY, USA). Using the culturing method (detailed in Section 4.2), we prepared cell suspensions of *S. epidermidis* and *P. acnes* cultures at bacterial density adjusted to 10^8^ CFU/mL. We spread bacterial suspensions on BHI or RCM agar plates for testing EOs against *S. epidermidis* or *P. acnes*, respectively. We immersed sterile filter-paper discs in 20 µL (EOs and DMSO) at either 0.7% or 1.4% and placed discs on the surface of the agar until dry; they were then incubated under appropriate conditions detailed for each bacterium above. We used two standard reference antibiotics clindamycin (2 µg/disc) and erythromycin (15 µg/disc) as reference controls, and we used DMSO as a negative control. We evaluated the antibacterial activity of each EO at each concentration by measuring the zone of inhibition diameter by Vernier’s caliper expressed in millimeters (mm). The assays were performed in triplicate and repeated as three independent experiments.

### 4.5. Determination of the MIC and MBC of the EOs

MIC and MBC of the EOs were determined using broth microdilution method in 96 well U shaped bottom microtiter plates in accordance with Clinical and Laboratory Standards Institute (CLSI) guidelines (2011) [38]. We dissolved EOs in sterilized DMSO at a final concentration of 7% (*v*/*v*), and then performed serial two-fold dilutions from 0.00875–0.56% (*v*/*v*) of the EOs. We prepared an inoculum of *S. epidermidis* and *P. acnes* in BHI broth and RCM, respectively, with OD_600nm_ adjusted to 0.5. We diluted the inocula to obtain a final turbidity in wells approximately 10^6^ CFU/mL. We incubated aliquots of 50 µL of bacteria and 50 µL of different concentration of each EO, at 25 °C for 24 h for *S. epidermidis* in aerobic conditions and 37 °C for 48 h for *P. acnes* bacteria under anaerobic conditions. We measured bacterial density changes using ELISA plate reader (InfiniteF50 Tecan-Sweden) at wavelength of 620 nm. We determined the lowest concentration of EO at which no bacterial density was detected as the MIC of the EO. We tested each EO at each concentration in triplicate and repeated the experiment three independent times. We used 50 µL of bacteria-free broth and 50 µL DMSO as negative control. For MBC determination, we inoculated 10 µL of mixtures of respective bacteria and EOs at different concentrations on agar plates (as detailed above) and determined bacterial counts expressed as CFU/mL after incubation at 37 °C at 24 and 48 h for *S. epidermidis* and *P. acnes*, respectively. We determined MBC as the lowest concentration of the EO at which incubated respective bacteria showed no detectable colonies on respective agar plates. To determine MBC, we tested each EO at each concentration in triplicate and repeated the experiment three independent times.

### 4.6. Minimum Biofilm Inhibitory Concentration (MBIC)

We determined MBIC for EOs against *S. epidermidis* biofilm using the microtiter plate method, as described previously [39], with some modifications. Briefly, we incubated 100 µL of *S. epidermidis* at 10^8^ CFU/mL in BHI–1% glucose (*w*/*v*) with 100 µL of the EO concentrations 0.00875–0.56% (*v*/*v*) at 25 °C for 24 h. Following incubation, we removed the contents of each well and gently rinsed the wells twice with 300 µL of PBS. We air dried the plate for 30 min and stained the formed biofilm with 0.1% (*w*/*v*) crystal violet (CV) for 30 min at room temperature. We then washed the excess CV from the plate with 200 mL of PBS per well, repeated washing three times and air-dried the plate. To measure the biofilm stained with CV, we solubilized CV using 95% (*v*/*v*) ethanol and measured the CV color intensity at OD_595nm_ using a Microplate reader (Infinite F50 Tecan). We determined MBIC for oregano, thyme, lemongrass, tea tree EOs, and thymol. We used BHI broth as a negative control and *S. epidermidis* cell culture without EOs as a positive control. We determined MBIC as the EO concentration at which the OD_595nm_ is equal to that of the negative control. Experiments for each EO concentration were performed in triplicate and the assay was repeated three independent times.

### 4.7. Determination of Kill Kinetics

Time-kill kinetics assay was performed for oregano EO against *S. epidermidis* and *P. acnes* as described previously [40]. Bacterial suspensions at a final concentration of 10^8^ CFU/mL were used as the initial inoculum. We assayed oregano EO killing time at concentrations of mg/mL (0.035%), mg/mL (0.07%), and mg/mL (0.14%) equivalent to 1, 2, and 4 MIC. We incubated different concentrations of oregano EO with the two bacterial cultures and measured killing capacity at 0, 1, 2, 4, 8, 12, and 24 h using broth micro-dilution method. At each time point, we used 50 µL of the assay solution to make ten-fold serial dilutions and performed viable counts on BHI and RCM agar plates for the respective bacteria. We incubated the agar plates at 25 °C for 24 h under aerobic conditions and 37 °C under anaerobic conditions for 48 h for each respective bacterium. We expressed viable count of each bacterium as CFU/mL. We used DMSO solvent and equivalent broth as negative controls. Each concentration of EO was assayed as triplicate and the entire assay was repeated three independent times.

### 4.8. Gas Chromatography-Mass Spectroscopy

We analyzed the chemical components of oregano and thyme EOs using gas chromatography-mass spectrometry (GC-MS) on a Shimadzu Model GC-17A gas chromatograph interfaced with a Shimadzu model QP-5000 mass spectrometer (Shimadzu, Kyoto, Japan). We separated volatiles on a DB5-MS column with specifications: 30 m length, 0.5 mm i.d., and 0.25 μm film (J&W Scientific, Santa Clara, CA, USA). We injected the EOs at a split ratio of 1:10 for the 30 s. We used the following operating conditions: Injector 220 °C, column oven 38 °C for 3 min, then programmed at a rate of 12 °C min^−1^ to 220 °C and kept for 2 min, His carrier gas at 1 mL min^−1^. We adjusted the transfer line and ion-source temperatures to 230 and 180 °C, respectively. We operated the HP quadrupole mass spectrometer in the electron ionization mode at 70 eV and set the scan range at *m*/*z* 40–500. We identified the volatile components using the procedures described previously [41]. We identified the resultant peaks after first de-convoluted using AMDIS software (www.amid.net) and we subsequently identified the compounds by their retention indices (RI) relative to n-alkanes (C6–C20), and by matching their mass spectra to the NIST, WILEY library database (>90% match) as well as to those of authentic standards when available.

### 4.9. Development of Nanoemulsion

A low energy method was used for preparation of oregano nanoemulsion [42], using 95% (*w*/*w*) of water, 5% (*w*/*w*) mixture of EO and Pluronic F127. The EO and surfactant mixture was prepared at a concentration of 4.5% of Pluronic F127 and the oil at 0.5% *w*/*w*. We added the defined amounts of oregano oil and Pluronic F127 and mixed by stirring at 800 rpm for 30 min. A stable nanoemulsion was formed by adding water drop wise at 3.5 mL/min flow rate while stirring at 800 rpm for 1 h. We stored the formed nanoemulsion at 25 °C and observed after 24 h and 1, 3, and 4 weeks of preparation. The droplet size and polydispersity index of the prepared nanoemulsion were measured using photon correlation spectroscopy (Zetasizer ZS, Malvern, UK). We expressed the mean diameter of the droplet size in μm and all measurements were made in triplicates.

### 4.10. In Vivo Antiacne Experiment

An initial preliminary experiment (*n* = 5 BALB/c mice) to examine the possible irritability of the oregano nanoemulsion was performed. In this preliminary experiment, we applied epicutanously 10 µL nanoemulsion (2 MIC against *P. acnes* as calculated by our in vitro assays) on ears of a group of healthy uninfected mice and observed over 5 days for any signs of inflammation.

In our in vivo experiments for assessment of oregano nanoemulsion efficacy, we used 45 BALB/c male mice divided into three groups (*n* = 5 mice/group/experiment). We measured mice ear thickness prior to injections, then, we injected mice the right ears intradermally with 20 µL PBS (healthy control ear) using Hamilton syringe 50 µL model 705 RN (Hamilton Co., Reno, NV, USA). We induced acne infection and inflammation by injecting the left ear with 20 µL of 10^8^ CFU *P. acnes* according to previously established protocol [43]. We observed the mice for microcomedones formation for 24–72 h and measured daily changes in ear thickness using electronic digital micrometer caliper (0–25 mm/0.001 mm). We considered appearance of microcomedones and the increase in mice ear thickness ≥10% as indicators of acne induction [43]. For infected mice ears, we applied epicutanously either 20 µL of 2 MIC oregano formulated nanoemulsion (test group), or 2% erythromycin (positive control), or no treatment (negative control) for 3 days. We recorded daily changes in mice ear thickness, weight, and took digital photographs of mice ears. At the end of the experiment, we excised mice ears and performed viable bacterial counts and histopathological assay.

We calculated percent inflammation post epicutaneous application of treatment for all the animal groups using the following formula:

$$\%\;\mathrm{inflammation}=\frac{\mathrm{ear}\;\mathrm{thickness}\;\left({}_{\mathrm{infected}\;\mathrm{ear}}\right)-\mathrm{ear}\;\mathrm{thickness}\;\left({}_{\mathrm{uninfected}\;\mathrm{ear}}\right)}{\mathrm{ear}\;\mathrm{thickness}\;\left({}_{\mathrm{infected}\;\mathrm{ear}}\right)}$$

We calculated the percentage inhibition of inflammation for all the animal groups using the following formula as described previously [14]:

$$\%\;\mathrm{inflammation}\;\mathrm{inhibition}=\frac{\%\mathrm{inhibition}\;\left({}_{\mathrm{control}}\right)-\%\mathrm{inhibition}\;\left({}_{\mathrm{treatment}}\right)}{\;\%\mathrm{inhibition}\;\left({}_{\mathrm{control}}\right)}$$

For histological examination, we preserved whole excised mice ears in 10% formalin-saline solution before preparing the histological sections using paraffin method technique. We dehydrated all sections in ascending grades of ethanol, cleared in xylene and then embedded in paraffin wax. We mounted transverse sections (4–5 micron, thickness) on glass slides and stained with hematoxylin and eosin (H&E) stains. We examined all sections for the evaluation of inflammatory response.

For in vivo assessment of anti-microbial activity of our nanoemulsion, we homogenized excised mice ears in PBS, then cultured the homogenates using plating serial dilutions method on RCM agar plates and counted *P. acnes* after 72 h of anaerobic incubation at 37 °C.

### 4.11. Statistical Analyses

All data were recorded in Excel worksheets (Microsoft Office 2010). Results were analyzed and plotted using both Microsoft Excel and Graph Pad Prism program (Version 6.1). The data presented is at least three independent experiments as mean ± standard deviation. For evaluation of statistical significance, *t*-test and one-way ANOVA test with Turkey’s multiple comparisons was used and considered *p* ≤ 0.05 significance level.

## Figures and Tables

**Table 1:** Minimal inhibitory concentration (MIC) of the seven tested EOs against *S. epidermidis* and *P. acnes*.

Essential Oils	MIC	
	*S. epidermidis* (mg/mL)	*P. acnes* (mg/mL)
Oregano	0.67	0.34
Thyme	1.30	0.65
Lemongrass	1.22	1.22
Tea tree	1.27	1.28
Lavender	2.52	2.52
Mentha	5.28	2.60
Chamomile	6.22	3.18

**Table 2:** Minimal bactericidal concentrations (MBC) of the selected EOs against *S. epidermidis* and *P. acnes*.

Essential Oils	MBC	
	*S. epidermidis* (mg/mL)	*P. acnes* (mg/mL)
Oregano	1.34	0.672
Thyme	2.60	1.30
Lemongrass	2.44	2.44
Tea tree	5.10	2.55

**Table 3:** Minimum biofilm inhibitory concentration (MBIC) of the selected EOs against *S. epidermidis*.

Essential Oils	MBIC
	mg/mL
Oregano	1.344
Thyme	2.60
Lemongrass	2.44
Tea tree	2.55

**Table 4:** GC-MS analysis of volatile compounds in oregano and thyme EOs.

Compound	Oregano EO Content %	Thyme EO Content %	Retention Time	Retention Index
Thymol ^a^	99.44	72.08	12.81	1280
*p*-cymene ^a^	0.2	25.18	8.79	1005
γ-terpinene	0.03	2.52	9.31	1038
α-Thujene	0.01	0.01	7.15	911
Cineole ^a^	0.06	−	8.96	1012
Thymol isomer	0.07	0.00	12.68	1274
Isocaryophyllene ^a^	0.04	−	14.34	1428
Carvacrol	−	0.07	12.67	1272

^a^ represents volatiles compared with authentic standard.

**Table 5:** MIC, MBC and MBIC of thymol against *S. epidermidis* and *P. acnes*.

Acne-Causing Bacteria	MIC (mg/mL)	MBC (mg/mL)	MBIC (mg/mL)
*S. epidermidis*	0.7	2.8	2.8
*P. acnes*	0.7	1.4	–

## Supplementary Materials

The following are available online. Table S1: Zone of inhibition (mm) against *S. epidermidis* and *P. acnes*; Figure S1: GC-MS chromatogram analysis of (a) oregano oil, (b) thyme oil; Figure S2: Size distribution by the intensity of the developed oregano nanoemulsion.
