# Supplementary material for: Origanum vulgare L. Essential Oil as a Potential Anti-Acne Topical Nanoemulsion—In Vitro and In Vivo Study
Source: Molecules. 2018 Aug 28;23(9):2164. doi: 10.3390/molecules23092164 (PMC6225355; doi:10.3390/molecules23092164)
Supplement: Supplementary file 1 [file molecules-23-02164-s001.pdf]

Supplementary Materials

Table S1. Zone of inhibition (mm) against *S. epidermidis and P. acnes.*

**Essential Oils**	***S. epidermidis***		***P. acnes***	
	**0.7% of EO**	**1.4% of EO**	**0.7% of EO**	**1.4% of EO**
Oregano	20.67±1.15	30.67±1.15	20.67±1.15	31.67±0.58
Thyme	16.67±1.15	21.67±1.53	16.70±1.15	23.30±0.58
Lemongrass	11.67±1.15	17.33±1.15	11.67±1.15	19.67±0.58
Tea tree	9.33±0.58	13.67±0.58	9.33±0.58	17.67±0.58
Mentha	8.00±0.50	8.23±0.21	7.80±0.53	9.67±0.58
Lavender	7.67±0.58	11.00±0.00	7.67±0.58	11.60±0.58
Chamomile	7.33±0.42	7.47±0.50	7.50±0.50	7.96±1.00
Clindamycin	15.33±0.58	18.00±0.50	14.00±0.50	20.00±0.35
Erythromycin	10.00±0.00	14.10±0.36	12.50±0.00	16.00±0.50
DMSO	7.33±0.58	7.10±0.16	7.00±0.00	7.00±0.00
Thymol	19.33±0.58	28.67±1.15	19.33±0.58	24.33±0.58

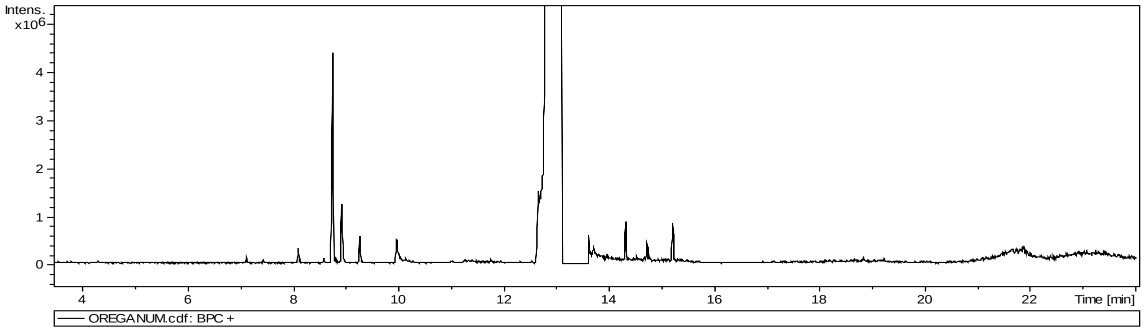

(a).

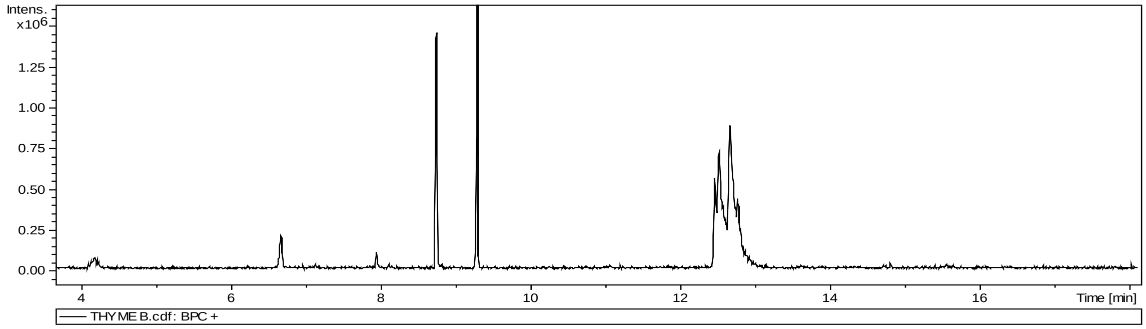

(b)

Figure S1. GC-MS analysis of (a) oregano oil and (b) thyme oil.

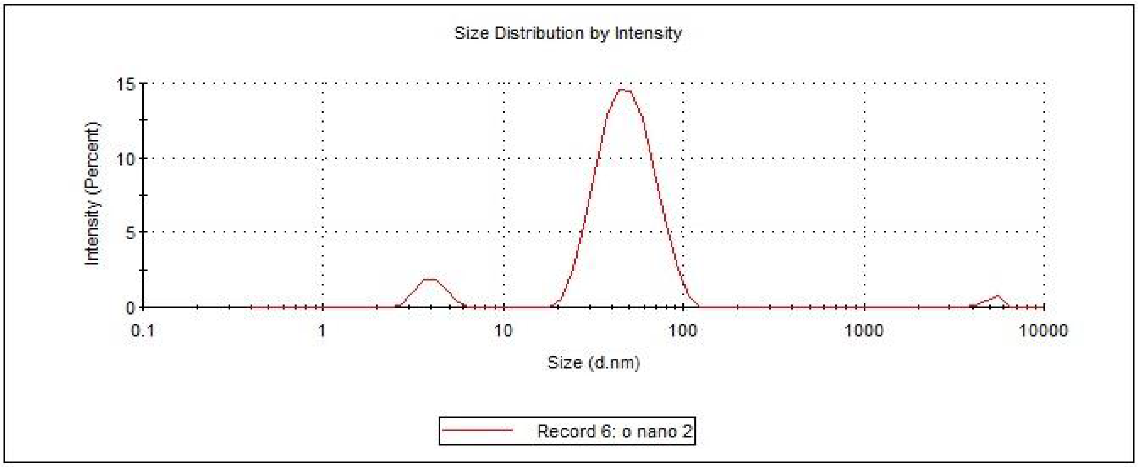

Figure S2. Size distribution by the intensity of the developed oregano nanoemulsion.
